# Microbial Biotechnology as a Catalyst for a Better and More Sustainable World

**DOI:** 10.1111/1751-7915.70196

**Published:** 2025-07-13

**Authors:** Carmen Michán, Juan L. Ramos

**Affiliations:** ^1^ Departamento de Bioquímica y Biología Molecular Campus de Excelencia Internacional Agroalimentario CeiA3, Universidad de Córdoba Córdoba Spain; ^2^ Estación Experimental del Zaidín, Consejo Superior de Investigaciones Científicas Granada Spain

**Keywords:** biotechnology, education, IMiLI, microbiology, UN SDGs

## Abstract

Education is our most powerful tool for transforming society and improving human well‐being. *Microbial Biotechnology* has previously addressed how we can contribute to enhance public understanding of microbiology in general, and microbial biotechnology in particular, along with their potential benefits. Here, we summarise several contributions in the Special issue ‘Societally‐relevant microbiology and outreach’.

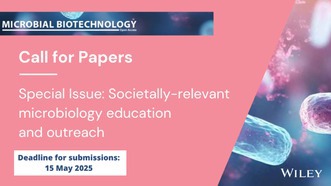

‘Science involves a pursuit of knowledge covering general truths or the operations of fundamental laws’ (Encyclopaedia Britannica). The hunt for knowledge by scientists enhances our understanding of the universe and can be used to improve our prospects for a better world. However, the question is better for whom? History and Science teach us that living organisms interact with one another but also with their non‐living environment. Thus, improving the lives of less‐privileged populations could be beneficious for the lives of all. Therefore, when focusing on human populations, addressing endemic issues such as chronic poverty and mental health challenges is essential for our well‐being and survival. In the Special Issue of *Microbial Biotechnology* ‘Societally‐Relevant Microbiology Education and Outreach’, several scientists explore how microbes can contribute to these goals.

First and above all, education. Education is our most powerful tool for transforming society and improving human well‐being. *Microbial Biotechnology* has previously addressed how we can contribute to enhancing public understanding of microbiology in general, and microbial biotechnology in particular, along with their potential benefits (Timmis et al. 2024, 2019; Timmis 2023; Ramos et al. 2025). But let us take this discussion several steps further.

## 1 How to Implement Microbiology Education?

### 1.1 Advancing Microbiology Education

Van Beek et al. (2024) have examined the challenges in microbiology education, particularly its limited visibility and over‐reliance on multi‐media teaching aids (MTAs). The International Microbiology Literacy Initiative (IMiLI) addresses these challenges by developing educational resources complemented by MTAs. Using a mixed‐methods approach, the study conducted literature reviews, developed multi‐media appraisal guidelines, piloted the guidelines and incorporated expert feedback. The finalised framework provides tools for audience‐specific multi‐media selection, evaluation and background resources. Broad adoption of these guidelines could improve multi‐media‐based learning outcomes, streamline resource selection and reduce disparities in microbiology education.

### 1.2 Community‐Based Learning in Microbiology

Valderrama et al. (2025) have presented their experience in service‐learning (S‐L) programmes that provide a bridge between academic knowledge and societal needs. For instance, the ‘Movies in Company for Preventing Diseases’ programme at the Complutense University of Madrid applies microbiology education to address public health issues, particularly in disadvantaged populations. The programme focuses on community needs, student engagement and social responsibility whilst fostering active participation beyond the university campus. By addressing health concerns and supporting underserved groups, it demonstrates how universities can effectively integrate learning with real‐world challenges. Its success serves as a model for addressing global health issues through education.

### 1.3 Fighting Antibiotic Resistance With Citizen Science

Gil‐Serna et al. (2025) have dealt with the issue of antibiotic resistance (AMR), one of the most critical global threats to human, animal and environmental health. Addressing AMR requires efforts in surveillance, research and education (Brüssow 2024a). The MicroMundo project, part of the Tiny Earth initiative in Spain and Portugal, adopts a service‐learning approach to promote AMR awareness and microbiology education. Gil‐Serna and colleagues present in this special issue ‘MicroMundo’, an initiative aimed to engage university and high school students in a citizen science project to isolate microorganisms from soils that may produce new antibiotics. Over 7 years, the initiative has expanded to 32 hubs across Spain and Portugal, involving thousands of students. This innovative project underscores the value of integrating education with scientific discovery to address global health challenges. This and other issues are being addressed in the global initiative ‘International Microbiology Literacy Initiative (IMiLI)’.

## 2 Education out of Educational Centres

### 2.1 Innovative Approaches to Microbiology/Microbiome Education

del Cerro‐Sánchez et al. (2025) have dealt with the concept of gamification; this is the use of game elements in non‐game contexts, which has proven to be a powerful tool in education, especially for engaging children in science. Inspired by Diane Ackerman's idea that play is essential to learning, educators are increasingly using gamified strategies to make complex subjects more accessible and enjoyable. This is particularly useful in microbiology, a subject often overlooked due to its abstract and invisible nature. Although most existing games focus on human health aspects of microbiology, a few innovative board games explore the environmental roles of microbes and their impact on climate change. However, there is still a lack of such tools in primary education. To address this gap, Del Cerro et al. explain the creation of a science outreach consortium from six Ibero‐American countries that launched a gamified programme in 2023. Targeting children aged 7–14 years in underprivileged areas, the initiative includes interactive talks, games and hands‐on experiments designed to teach environmental microbiology and its connection to climate change. The programme aims to evaluate its effectiveness in schools across Latin America and ultimately provide educators with tools to better teach these critical topics.

Schweitzer et al. (2024) have highlighted recent research on how daily habits and lifestyle choices shape the human microbiome, influencing health and disease. To educate a broad audience, the authors have developed the video game *Tiny Biome Tales*, based on peer‐reviewed studies. This interactive game explores the impact of lifestyle on the microbiome across various life stages. A scientific assessment showed significant knowledge improvement amongst players. With its innovative ‘gamified review’ format, the game offers flexibility for updates and new content, positioning it as a powerful tool for disseminating microbiome research.

Furthering microbiome education, the CIFAR initiative has developed three public health curricula focused on breastfeeding, antibiotic use and diet. Melby et al. (2025) have described the ‘Human & the Microbiome’ (HMB) programme, which aims to bridge scientists and the general public via health professionals. These professionals will receive free educational materials covering the three core topics. The benefits of breastfeeding, highlighted by Schwab (2022), and Massi and Stewart (2024), include infection protection, immune system support and reduced long‐term risk of chronic diseases. CIFAR emphasises that both breastfeeding and diet significantly influence the human microbiome, with poor dietary habits contributing to cardiovascular issues. The overarching goal was to foster societal impact by illustrating the essential role of microbes in health and encouraging daily practices that prevent child mortality, chronic illness and antibiotic resistance.

### 2.2 Science Communication and Global Challenges

Yarzábal Rodríguez and Batista‐García (2025) have described that in an age of rampant misinformation, particularly during crises, such as the COVID‐19 pandemic, science communicators play a crucial role. Researchers often struggle to convey complex ideas to the public, but combining science and art has proven effective in expanding outreach. In microbiology, this synergy can educate communities and enhance awareness, especially amongst students. Since Alexander Fleming's time, microorganisms have fascinated artists, and art has helped demystify microbes, making them approachable and fostering public engagement with science. Rodríguez et al. (2025) have shared experiences in using art to teach microbiology and highlight the need to promote scientific literacy across all ages and communities, particularly underserved groups. These efforts align with the goals of the International Microbiology Literacy Initiative (IMiLI) (Timmis et al. 2019).

## 3 Education for All

### 3.1 Microbiome and Criminal Behaviour

Logan et al. (2025) have put forward the potential of our microbiome to influence our social behaviour and the consequences for criminal justice. Previous social and educational backgrounds have been linked to behaviour and used as defences for criminal responsibility. Several recent studies have proved the connection between gut microorganisms and behaviour by influencing brain functions, for example, the production of neurotransmissions, the absorption of nutrients or the functioning of the blood–brain barrier. Furthermore, the presence of intestinal and oral microbes has been associated with mental disorders and aggressive or addictive conduct. Altogether, the knowledge of the human microbiome opens new ways of prevention, treatment and rehabilitation of criminals.

### 3.2 Science Outreach for Marginalised Groups

de Lorenzo (2024) has described a science outreach initiative by the Spanish National Research Council (CSIC) and NGO *Solidarios para el Desarrollo*, which educates prison inmates about microbiology. The programme highlights the Earth's microbial ecosystems, human microbiomes and microbial biotechnology through simple metaphors and engaging examples. By fostering curiosity and critical thinking, the initiative helps inmates endure their sentences and prepares them for reintegration into society. Despite facing challenges, the programme demonstrates the transformative power of bringing science to marginalised groups.

## 4 Education and Science Today

### 4.1 Sustainability and Education

Paoli (2024) has explored how profit‐driven social habits and economic systems hinder progress toward sustainable development. Consumerism and technological advancements have distanced many communities from nature, exacerbating global challenges, such as the climate crisis and ecosystem degradation. The author argues that education for sustainable development and science‐based decision‐making provide essential opportunities for driving positive change and building a sustainable future.

### 4.2 The Role of AI in Public Health

de Carvalho et al. (2025) have tackled a relevant aspect, often overlooked, that of infectious diseases, which remain a leading cause of pandemics, creating challenges in predicting outbreaks, detecting variants, developing drugs and combating misinformation. Artificial intelligence (AI) offers tools to address these issues, as demonstrated during the COVID‐19 pandemic (Brüssow 2024b). However, equitable and responsible AI use is essential, requiring global cooperation to establish shared regulations and guidelines. The Global South AI for Pandemic & Epidemic Preparedness & Response Network (AI4PEP) has launched 16 projects across several countries to enhance public health systems. Its branches in Latin America and the Caribbean (LAC) aim to combat infectious diseases in low‐ and middle‐income countries, leveraging AI to improve prevention, preparedness and response. This initiative demonstrates the potential of AI to benefit community health and well‐being in under‐resourced regions.

### 4.3 Microbial Technologies for Global Challenges

Timmis and Hallsworth (2024) have emphasised the vast potential of microbial technologies to address humanitarian and environmental challenges, particularly in sustainability and low‐resource settings. These technologies support healthcare, agriculture, pollution reduction and ecosystem restoration. However, their current applications remain inadequate. The authors urge microbiologists to lead efforts in promoting and deploying these technologies by collaborating with scientists, policy makers, industries and the public. Effective partnerships and institutional support are vital, especially as humanity and the biosphere face unprecedented threats. Immediate action by microbiologists and their societies is critical to harness the life‐saving and planet‐healing potential of microbial technologies.

### 4.4 Microbiome Applications and Challenges

Kostic et al. (2024) have discussed the growing significance of microbiomes in human, animal, and plant health, food production, and environmental management, driven by substantial R&D investments and market expansion. Despite this progress, the lack of a unified definition for microbiome applications creates obstacles in communication, documentation, commercialisation and public acceptance. To address these issues, the authors highlight two established applications and propose criteria to define microbiome applications. These criteria aim to enhance communication, establish robust regulatory frameworks and build trust amongst stakeholders.

### 4.5 Science Reliability

Timmis et al. (2025) address one of the current main drawbacks in reliability which is the proliferation of predatory journals. Their sole economic purpose and lack of scientific rigour cause general distrust in scientific publications. Science is always based on previous advances; thus, publications should be based on rigour, reproducibility and unbiased interpretation of results. Main journal rankings and indexes need to establish clear codes to avoid this practises. Furthermore, we scientists have a moral duty of pursuing these standards. Moreover, most of us are supported by public funding and we should be committed to public benefits. The ‘publish or perish’ mantra imposed by many academic systems needs to be rethought to encourage quality instead of quantity. Learned societies and journal editorial teams should play a pivotal role in doing so.

## 5 Concluding Remarks

All these initiatives could be summarised in three different objectives.

### 5.1 To Advance in Microbiology Education for Global Impact

Advancing microbiology education requires inclusive and innovative strategies that connect innovative learning with real‐world outcomes. The IMiLI supports this by offering multi‐media tools and educational guidelines to enhance learning through regional hubs, such as IMiLI‐EAH (East‐Asia Headquarters, https://www.imili‐eah.com/), IMiLI‐CAC (Central America and the Caribbean, https://www.chavarrialab.com/imilicac), Spain (https//grupos.eez.csic.es/imili/) and IMiLI‐SAC (South Asia Centre, https://imili.org/) (Ramos et al. 2025). Programmes such as CIFAR, gamified science programmes, Valderrama's service‐learning and the MicroMundo citizen science project demonstrate how education can address environmental and public health concerns, such as antibiotic resistance, diet and microbiota. Together, these efforts are building a globally relevant and socially responsible model for training future scientists.

### 5.2 To Use Microbiology Education as a Tool for Empowerment

Education in microbiology holds transformative potential, particularly for marginalised populations. Research linking the human microbiome to behaviour opens up new pathways for rehabilitation, including in the criminal justice system. At the same time, science outreach programmes, such as microbiology education in Spanish prisons, show how accessible and engaging microbiology education can foster curiosity, resilience and reintegration. These efforts emphasise the importance of inclusive science education as a powerful driver of empowerment and social change.

### 5.3 To Build an Informed and Resilient Society

In the face of global challenges—climate change, pandemics and misinformation—education rooted in science, ethics and international collaboration is essential. Promoting sustainability, deploying responsible AI in public health and harnessing microbial technologies are crucial to building a healthier and more equitable future. These efforts should be based on scientific integrity and clarity. Building a resilient, informed society depends on rethinking education, ensuring research quality and empowering global cooperation.

## Author Contributions

**Carmen Michán:** conceptualization, writing – original draft, writing – review and editing. **Juan L. Ramos:** conceptualization, writing – original draft, writing – review and editing.

## Conflicts of Interest

The authors declare no conflicts of interest.

## Data Availability

Data sharing not applicable to this article as no datasets were generated or analysed during the current study.
